# Dataset on the physical characterization of biopolymer coated magnetic nanoparticles

**DOI:** 10.1016/j.dib.2016.11.038

**Published:** 2016-11-17

**Authors:** Shazia Bano, Muhammad Afzal

**Affiliations:** a Department of Physics, The Islamia University of Bahawalpur, Pakistan; b Nanosciences and Technology Department (NSTD), National Centre for Physics (NCP), Islamabad, Pakistan

**Keywords:** Biopolymer

## Abstract

The data presented in this article is related to the research article entitled “Paclitaxel loaded magnetic nanocomposites with folate modified chitosan/carboxymethyl surface; a vehicle for imaging and targeted drug delivery” (S. Bano, M. Afzal, M.M. Waraich, K. Alamgir, S. Nazir, 2016) [1]. It contains the absorbance spectra, band gap energies of pure nickel-ferrite nano cores (NFs), and calibration curve of Paclitaxel. Thermal stability analysis of pure NFs, chitosan (CS) and carboxymethyl cellulose (CMC)-conjugated NFs samples is also included in the data.

**Specifications Table**

**Table:** 

Subject area	Physics
More specific subject area	Medical Physics and Nanomedicine
Type of data	Graphs
How data was acquired	UV/VIS/NIR spectrometer (Perkin Elmer) X-ray powder diffractometer (Bruker D8 Advance) in a 2*θ* range of 20°–70°. Rutherford Backscattering Spectroscopy (RBS) Thermogravimetric and Differential Thermal Analyzer (TG/DTA), Perkin Elmer (Pyris 1).
Data format	filtered
Experimental factors	For XRD, DRS and TGA dry powder of sample was used.
Experimental features	UV/VIS/NIR spectra were recorded in an optical-quality quartz cuvette with a 1 cm path length at room temperature. TGA was performed at a heating rate of 10 ^°^C/min.
Data source location	National Centre for Physics, Islamabad, Pakistan CRL, University of Peshawar, Pakistan
Data accessibility	Data is within this article

**Value of the data**

• Beneficial to develop a single combinatorial approach.
• Optical, elemental and thermal properties help to understand the behavior of metallic nanoparticles in different environment to be used for different applications.
• Photodynamic potential of nanoconstruct can be predict.

## 1 Data

The dataset of this article contained information on the XRD patterns, absorbance and diffuse reflectance spectra (DRS), of the NiFe2O4 nanocrystals. Thermal stability analysis of pure NFs, CS and CMC-conjugated NFs samples and standard calibration curve for known sample of Paclitaxel is also included in the data.

## 2 Experimental design, materials and methods

We combined the merits of NiFe_2_O_4_ nanoparticles and naturally occurring polysaccharides chitosan and carboxymethyl cellulose with bovine serum albumin to fabricate Paclitaxel (PTX) containing bionanocomposites [1]. Nanoparticles were synthesized via thermal decomposition of metal precursor and were subjected to optical, elemental and thermal analysis after hydrophilic surface modification.

**Fig. 1:**
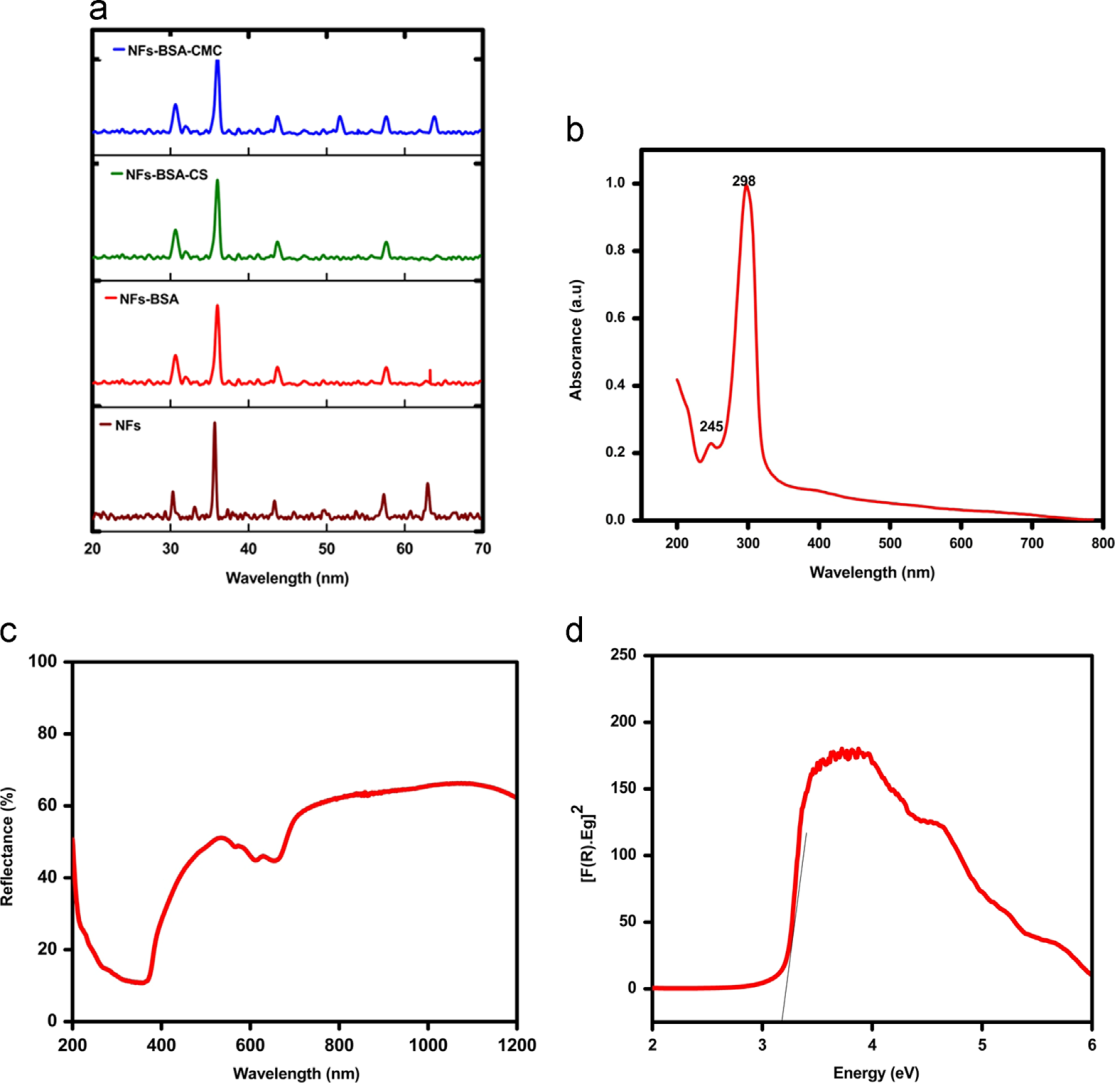
**a** XRD patterns of NFs, NFs-BSA, NFs-BSA-CS and (NFs-BSA-CMC. **b:** UV/VIS/NIR spectrum of NFs. **c:** Diffused reflectance spectrum of NFs. **d:** Band gap energies of NFs.

UV/VIS absorption spectrum of NFs (Fig. 1b) shows the high intensity peak intensity at 298 nm and a small peak at 245 nm the characteristics wavelengths. Characteristic absorbance in range of 400 nm–600 nm obtained by DRS (Fig. 1c) covering the most of visible region. Band gap energies may be estimated from the intercept of tangent drawn to the plot of Kubelka–Munk function vs energy in electron volts (eV) and was found 3.2 eV and may have potential application in the visible-light driven photodynamic therapy.

**Fig. 2:**
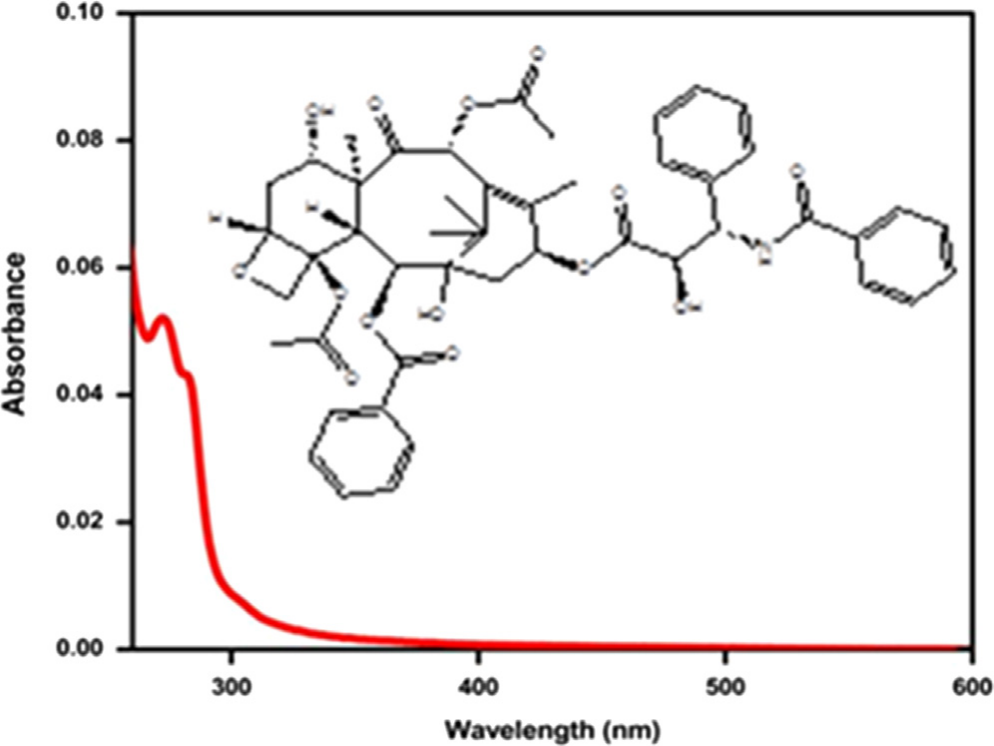
Structure and absorbance spectrum of Paclitaxel (Molecular weight 853.906 g/mol).

**Fig. 3:**
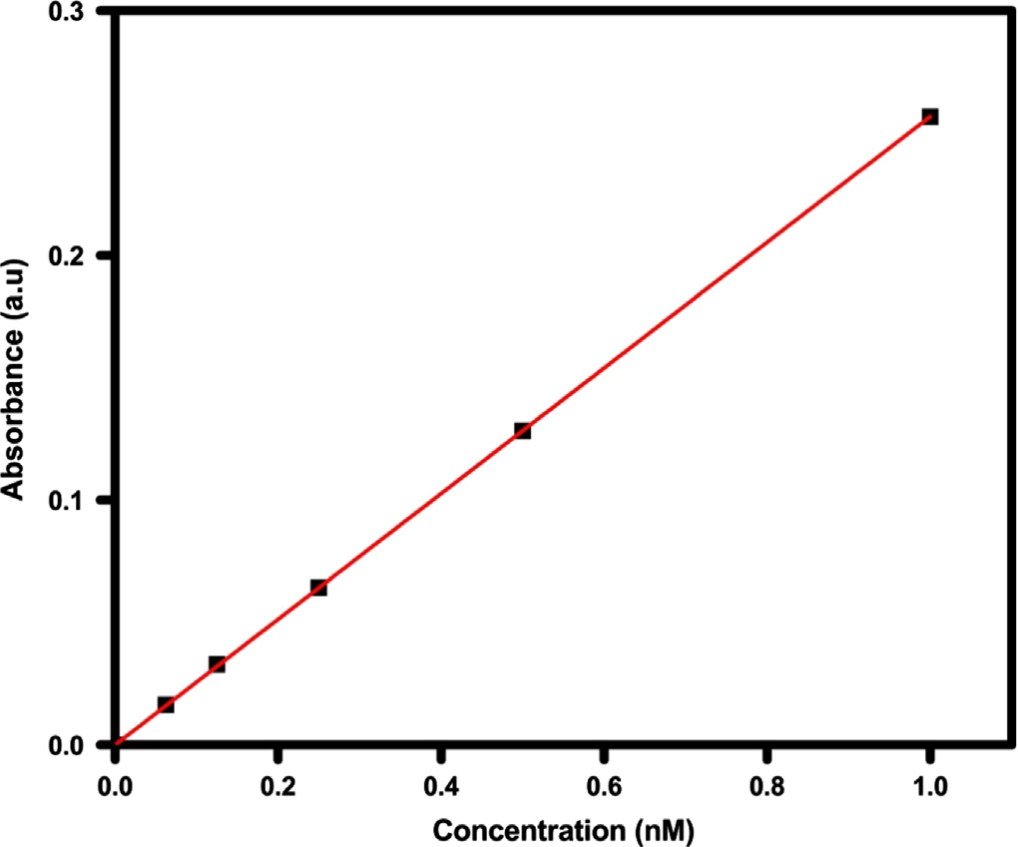
Standard curve of Paclitaxel.

We used BSA as a matrix to load PTX. The drug is approved for the treatment of ovarian, lungs, bladder, breast and other types of solid tumors. The structure and absorbance spectrum of PTX is shown in Fig. 2. Standard calibration curve obtained at 277 nm of known concentrations of the drug (Fig. 3) and superparamagnetic NiFe_2_O_4_ nanocores. Bonding of biopolymers to the NFs and their drug loaded conjugates was confirmed by FT-IR spectrometer.

**Fig. 4:**
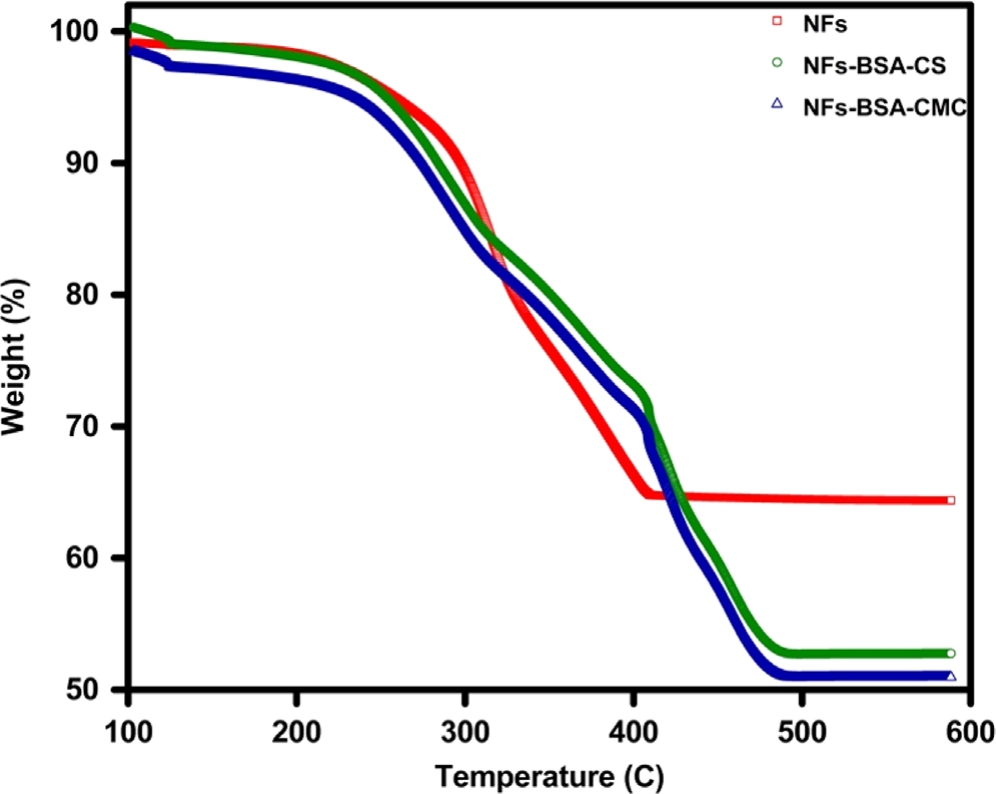
TGA curves of pure NFs synthesized by thermolysis, CS and CMC-conjugated NFs samples.

Thermal stability analysis was investigated by a Thermogravimetric and Differential Thermal Analyzer (TG/DTA); Perkin Elmer (Pyris 1) at a heating rate of 10 °C/min. Decomposition takes place in three regions over the range of room temperature to 480 °C, with a weight loss of 3.4% for the pure NFs and 4.7% for the NFs-BSA–CMC (Fig. 4).
